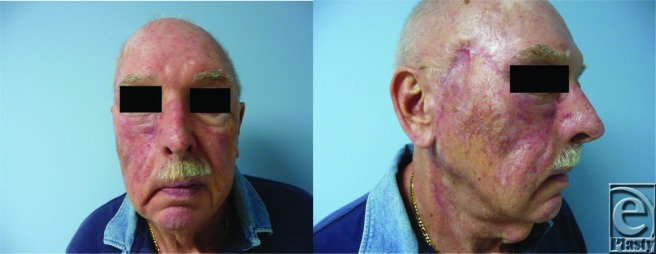# Reconstructing Zone 1 Cheek Defects

**Published:** 2013-01-28

**Authors:** Mark Nicolau, Mark S. Granick

**Affiliations:** Division of Plastic Surgery, New Jersey Medical School—University of Medicine and Dentistry of New Jersey, Newark

**Figure:**
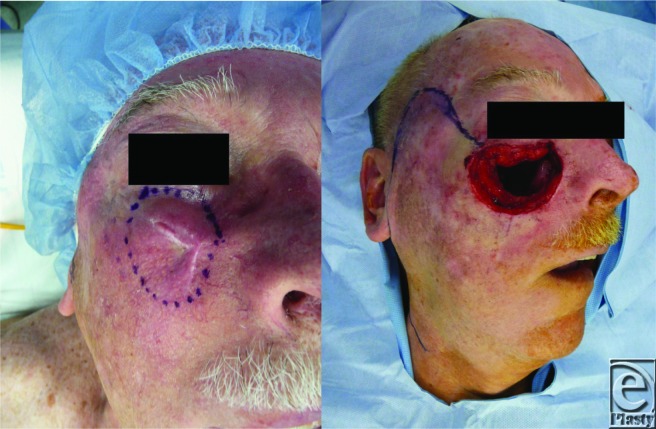


## DESCRIPTION

This patient is a 76-year-old man with medical history of diabetes mellitus, hypertension, and multiple squamous cell skin cancers. Four years prior, he underwent dermatographic resection of a squamous cell carcinoma of the right cheek that had involved the right facial nerve. Two years later, the lesion recurred. Radical resection with control of free margins was performed by the head and neck oncology service. The underlying maxilla and the infraorbital nerve were involved in the resection leaving the above defect.

## QUESTIONS

1. **Describe the defect and the zones of the cheek.**
2. **What options are available for the reconstruction of this wound?**
3. **What are the major potential complications of reconstruction of this defect, and how can it be avoided?**

## DISCUSSION

The patient's tumor recurred with the deep margin extending to the anterior maxillary wall, including a portion of the infraorbital nerve. To ensure free margins, the overlying soft tissue, the maxilla, and the most accessible part of the infraorbital nerve were resected. It is a full-thickness defect involving subcutaneous fat, superficial muscular aponeurotic system (SMAS), underlying musculofascial system, and the anterior maxillary wall. Zide et al^1^ have divided the cheek into overlapping zones that encompass the esthetic unit. Zone 1 includes the suborbital and malar regions, zone 2 encompasses the temporal and preauricular area, and zone 3 contains the perioral and mandibular regions. This defect is located in zone 1.

Reconstructive efforts for zone 1 defects have been previously described. Small defects can be closed primarily along the relaxed skin tension lines. Larger defects can either be skin grafted over or covered by local or free tissue transfers. If skin grafts are to be used, full-thickness grafts should be made using templates to ensure adequate size and shape. The best donor sites include areas with similar skin tone and thickness; preferred areas include the preauricular, postauricular, and clavicular regions, all comprising an area termed the “blush zone.” Defect depths less than 5 mm have the best esthetic result. If the defect does not permit skin grafting, skin transposition flaps can be used. These flaps have been widely described and utilize the local tissue and blood supply to reconstruct the defect. For lesions smaller than 4 cm, rhomboid, v-y advancement, and bilobed flaps can be used. Rhomboid flaps allow for a smaller donor site to be used, due to the ability of redistribution of tension around the suture line. Bilobed flaps are used for larger and more central defects and utilize both pre- and postauricular donor sites; although the flap divides tension between 2 advancement flaps, it leaves a much larger scar. No matter which skin transposition flap is used, the idea is to design a flap which utilizes skin laxity around the natural tension lines of the face, as well as providing primary closure of the donor site without compromising the mobile landmarks of the face, such as the lateral canthus, nasal alae, nasolabial folds, and the commissures of the mouth.

Cervicofacial flaps are the mainstay for defects larger than 4 cm. These flaps are quite versatile and many modifications have been described. These flaps can be extended down even into the upper chest if more coverage is needed. With these flaps, other procedure, such as canthopexy, can be performed because of the same area being exposed.

Boutros and Zide have described an “angle rotational flap” which utilizes pre- and postauricular donor sites to cover cheek defects. The tissue is moved both superiorly and medially, moving the postauricular tissue to the preauricular donor site, and the preauricular tissue is moved to cover the defect.

As compared with the previously mentioned flaps, the angle rotational flap provides minimal scar in the neck and chest region. The flap is designed so that there is no cervical involvement, and that the donor tissue is advanced from the postauricular area superiorly and medially to cover medial cheek defects.

Although these reconstruction efforts are widely used, surgeons must be aware of the possible side effects when entering this area of the face. Similar to facelifts, cervicofacial flaps, when dissected out, can potentially damage the branches of the facial nerve, as well as the greater auricular nerve. Staying above the SMAS can decrease damaging the facial nerve branches. This is because the facial nerve runs deep the SMAS, in the deep fascial plane. Different areas of the face, however, differ in thickness with regard to the layers of the face. This must be kept in mind to ensure decreasing damage to the deeper structures of the face. The greater auricular nerve innervates the cutaneous portions overlying the parotid gland, the mastoid process, and the surface of the ear. Because of its superficial location, about 6.5 cm inferior to the tragus on the sternocleidomastoid, it also can be damaged during a cervicofacial flap dissection.

Reconstruction of zone 1 defects can lead to ectropion once the flap is healed, due to the natural contracture of healing wounds. Technical consideration in cheek reconstructions is necessary for the avoidance of this late complication. During the setting of the flap, sutures can be anchored to the periosteum of the zygomatic arch and inferior orbital rim to decrease ectropion. A lateral canthopexy can also be performed and is often done to prevent ectropion. This patient presented with involvement of the facial nerve, presenting with ectropion and facial droop on the right. To prevent further ectropion and facial droop, a lateral canthopexy and a temporalis fascial sling to the commissure of the mouth were performed prior to closure of the flap.

## Figures and Tables

**Figure:**